# The relative stability of trpzip1 and its mutants determined by computation and experiment†

**DOI:** 10.1039/d0ra00920b

**Published:** 2020-02-12

**Authors:** Hailey R. Bureau, Stephen Quirk, Rigoberto Hernandez

**Affiliations:** Department of Chemistry, Johns Hopkins University Baltimore MD 21218 USA r.hernandez@jhu.edu; Kimberly-Clark Corporation Atlanta GA 30076-2199 USA

## Abstract

Six mutants of the tryptophan zipper peptide trpzip1 have been computationally and experimentally characterized. We determine the varying roles in secondary structure stability of specific residues through a mutation assay. Four of the mutations directly effect the Trp–Trp interactions and two of the mutations target the salt bridge between Glu5 and Lys8. CD spectra and thermal unfolding are used to determine the secondary structure and stability of the mutants compared to the wildtype peptide. Adaptive steered molecular dynamics has been used to obtain the energetics of the unfolding pathways of the mutations. The hydrogen bonding patterns and side-chain interactions over the course of unfolding have also been calculated and compared to wildtype trpzip1. The key finding from this work is the importance of a stabilizing non-native salt bridge pair present in the K8L mutation.

## Abstract 2

The single-point mutations of tprzip1 are indicated at left, and their relative energetics are compared at right.

## Graphical abstract

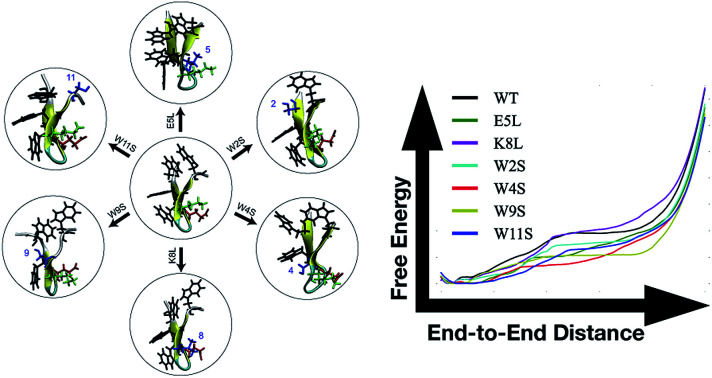

## I. Introduction

The stability of an isolated biomolecule is dictated by numerous factors including amino acid sequence, specific side chain interactions, hydrogen bonding effects, hydrophobic packing, backbone strain, and solvent environment. It is the interplay of these factors which is important; but, it is often difficult to obtain a quantitative understanding of the combination of those effects. Computational studies, particularly molecular dynamics (MD), lend a different perspective to this challenging problem because noncovalent intermolecular interactions can be readily observed and analyzed on the single-molecule scale. In this work, we explore the use of single-point mutations to reveal the relative stability and structural propensities as a peptide or protein is pulled apart using complementary experimental and computational approaches.

We focus on the β-hairpin motif because it has been a frequent target for computational and experimental biophysical studies that probe such effects.^1^ They are suitable because they are often small, adopt a specific native fold, and are stable. An isolated peptide motif, such as the β-hairpin, provides a system in which the local environment, such as solvent, can be specifically controlled. Typical system sizes remain small enough to model computationally but large enough to be of general interest to the community. To be specific, we take advantage of the family of small, stable β-hairpin peptides absent of disulfide linkages designed by Starovasnik and coworkers^2^ known as tryptophan zippers (trpzips). The smallest of those peptides, trpzip1 (PDB 1LE0), is the target of this paper. There have been several studies involving the various factors controlling the behavior and stability of trpzips.^3^ Notably, trpzip3 has been seen to possess anti-aggregation properties when introduced to two different amyloid-β systems.^4^

In the β-hairpin motif, cross-strand salt bridges have been seen to provide stabilization of the folding pathway and the native structure.^5,6^ However, this stabilization is very sensitive to the selected system, and is dependent on context and environment. For example, in a study involving several different β-hairpins of various lengths, Chen and coworkers^7^ concluded that Glu–Lys pairs are more favorable than Asp–Lys pairs due to side chain length matching. They found that the lengthening by one carbon atom (as in Glu) adds significant electron donating character to the carboxylate group by decreasing the withdrawing character of the backbone; creating an enhanced electrostatic interaction. However, a study by Kiehna and Waters^8^ determined that the salt bridge contribution was negligible when compared to Phe–Phe interactions. Hydrogen bonds are also important interactions within peptides.^9^ They have been seen to contribute to the stability of both parallel and antiparallel β-sheet structures,^10^ and to aggregation behavior and higher-level protein structure formation between β-sheets.^10^ Meanwhile, Zhao and Wu^11^ performed several theoretical studies on helices and β-hairpins in which they observed no cooperative enthalpic contributions from hydrogen bonds.^12^ Sauer and coworkers^13^ mutated a buried salt bridge triad of residues to hydrophobic residues in an Arc repressor protein, known to play a critical role in Arc repressor function. Interestingly, the hydrophobic mutant exhibited enhanced stabilization, implying that the charged residues were not essential to stability. Maynard, *et al.*^14^ performed a thermodynamic analysis using a combination of NMR and circular dichroism methods on the folding of a designed 16-residue analogue of the MET repressor protein dimer. They concluded that β-hairpin folding is driven by hydrophobic effects, not hydrogen bonding as had been previously suggested. This is in contrast to a trpzip mutation study by Keiderling and coworkers,^3^ in which they concluded that turn stability and hydrophobic packing are the main forces controlling the stability of β-hairpins. Ionizable residues in hydrophobic environments have previously been shown to play a significant role in the dynamics of peptides particularly with respect to the solvation around titratable residue pairs.^15^ Thus there remains no clear winner as to which interaction, or combination of interactions, leads to the stability of a given protein.

**Fig. 1:**
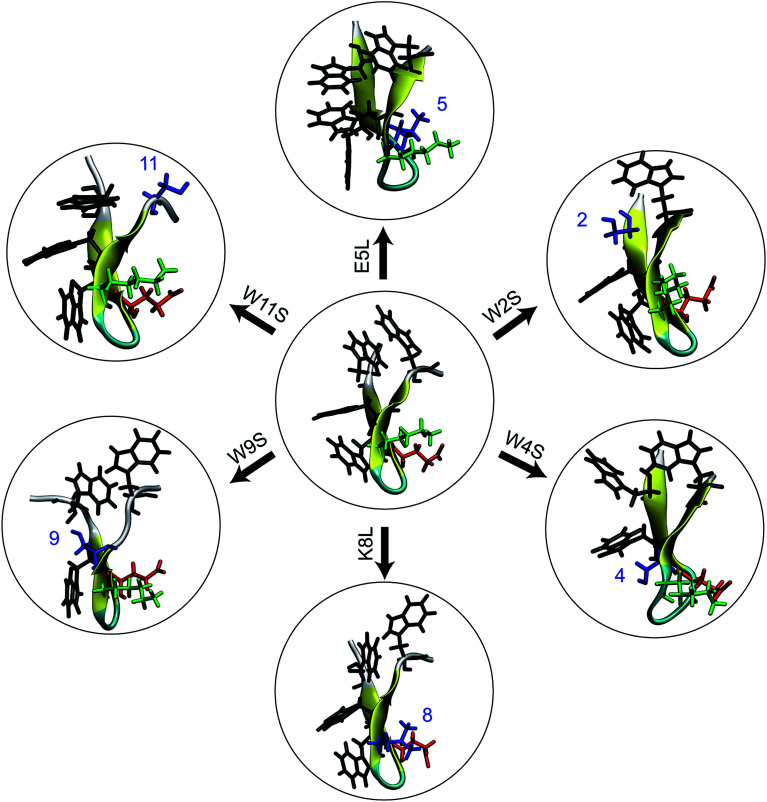
An illustration of the seven peptides studied in this work. At the center is the WT trpzip1. The six peptides around the WT are the mutations and are labeled over the arrow. On the WT peptide, the residue side chains in black, green, and red are the tryptophans (at positions 2, 4, 9, and 11), lysine-8, and glutamic acid-5, respectively. In the mutant peptides surrounding the WT peptide, the mutated residue sidechains are shown in blue. All structures are obtained at the end of a constrained relaxation of the solvated protein holding the terminal C_α_s at the positions of the corresponding equilibrated peptide in vacuum. The structures serve as the initial conditions for the respective ASMD simulations.

In this work, we combine experimental and computational techniques to determine and compare the stability of trpzip1 and six mutations so as to help resolve this general question with respect to this particular and important peptide. The mutations shown in Fig. 1 were selected specifically so as to destabilize specific native amino acid contacts and/or to disrupt specific side chain interactions. We use circular dichroism (CD) spectra and thermal unfolding to experimentally determine the structure and stability of the peptides. Using simulations, we determine the single-molecule energetics, hydrogen bonding pattern, and side-chain interactions for the mechanical unfolding of the trpzip1 and six systematic point mutations. Specifically, we use adaptive steered molecular dynamics (ASMD)^16–18^ to obtain the potential of mean force (PMF) and other observables along the pulled unfolding pathway not just for the wild type protein as in our previous work,^19^ but also for the family of mutants addressed in the experiments presented here.

The paper is organized as follows: in Section II, we review the computational approaches used to simulate the unfolding of the peptides. In Section III, we present the experimental methods we used to investigate the stability of the peptides, namely, circular dichroism and thermal unfolding. We discuss our results beginning with the experimental results in Section IVA. The comparison of the potentials of mean force (PMF) obtained computationally are presented in Section IVB. The hydrogen bonding trends are presented in Section IVC, and the interaction energies of specific residue pairs are surmised and contrasted for each mutant in Section IVD. This work thus provides a detailed comparison of the structure and stability of six trpzip1 mutations offering additional perspective on the degree to which different interactions contribute to their stability.

## II. Computational methods

### A. Mutations

The primary sequence of wildtype (WT) trpzip1 is: SWTWEGNKWTWK. For the simulations, six single-point mutants were built using the VMD plugin Mutator:^20^ E5L, K8L, W2S, W4S, W9S, and W11S. The mutants are denoted using the original residue, residue number, and new residue. The equilibrated WT trpzip1 structure was used as the base for the mutations. The set of six mutants was chosen in order to probe the hydrophobic core and the Glu–Lys salt bridge. Leucine was chosen as the replacement residue for the salt bridge residues because of its propensity as a β-sheet forming amino acid^21–23^ Despite the small size of the peptides, the single-point mutations did not cause any significant structural changes in the backbone, and the secondary structure remained that of a stable β-hairpin during the 1 ns relaxation of the peptides in solvent. This was observed through use of the VMD plugin Timeline for secondary structure analysis of the relaxation of each peptide. We refer to these relaxations as “equilibrations” throughout the text, providing details or the procedure in Section IID, in the sense that the structures reached local equilibria under the constraint of the distance between the terminal C_α_ set to *r*_ee_.

### B. ASMD

The Adaptive Steered Molecular Dynamics (ASMD) method, which was previously developed^16^ and benchmarked for vacuum,^24^ implicit solvent,^25^ and explicit solvent^17^ conditions for the α-helical peptide Ala_10_, was used for calculating the PMFs of the six β-hairpin peptides. This method overcomes the sampling problems previously observed in standard Steered Molecular Dynamics (SMD) simulations.^26,27^ It is competitive with state-of-the-art approaches being advanced by the community such as those employed in ref. 28–32. In ASMD, the overall reaction coordinate is divided into *n*_s_ stages, and the PMF is calculated in each stage. ASMD has also been used to steer a ligand through a protein active site.^33^

In this work, the reaction coordinate is the length of the end-to-end distance, *r*_ee_, of the protein. The PMF calculation is carried out using Jarzynski's equality, as was previously implemented in conjunction with simulation.^26,34^ The calculation of the PMF is obtained for a specific position *r*_ee_(*t*) within the set (*r*_ee,*j*_, *r*_ee,*j*+1_). The average work for each stage is obtained through the Jarzynski equality as1
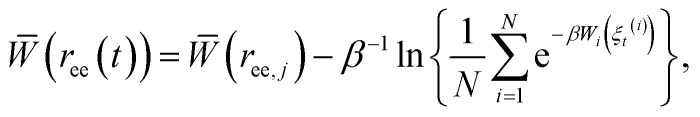
where *ξ*^(*i*)^ is the *i*^th^ trajectory in the nonequilibrium ensemble stretched from *r*_ee,*j*_. To choose the starting coordinates for the next stage of the calculation, the final value of the work for each trajectory is compared to the value obtained from the Jarzynski average. The trajectory with the closest work value to this average is chosen as the starting structure for the next stage. The final coordinates of this trajectory are used as the starting point from which the ensemble of structures for the next stage is generated. In the next stage, the velocities for each trajectory are randomized using the Maxwell–Boltzmann distribution.

### C. Observables

In addition to the energetic comparison of the peptides, the hydrogen bonds and specific residue pair interactions have been calculated and compared in Sections IVC and D, respectively. These observables are calculated for the peptides along the unfolding pathway. The average of the observables are obtained using in-house python scripts in conjunction with VMD Plugins HBonds and NAMDEnergy.^20^ The hydrogen bonds of a given configuration are counted when the donor and acceptor atoms from either backbone or side chains are within 3.0 Å of each other and when the angle formed between them is less than or equal to 40°.

They are also calculated for the two complementary cases in which the hydrogens are intrapeptide, and between the peptide and water, respectively. The interaction energies between the following residues are calculated and compared within each peptide: 2–11, 4–9, 5–7, 5–8, 5–12, and 8–10.

Each observable is weighted using the work associated with an individual nonequilibrium work trajectory.^24,35,36^ The averages for the hydrogen bonds are obtained as follows: for two separate groups S_1_ and S_2_ of selected atoms in 
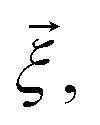
 the average number of hydrogen bonds can be obtained as2a
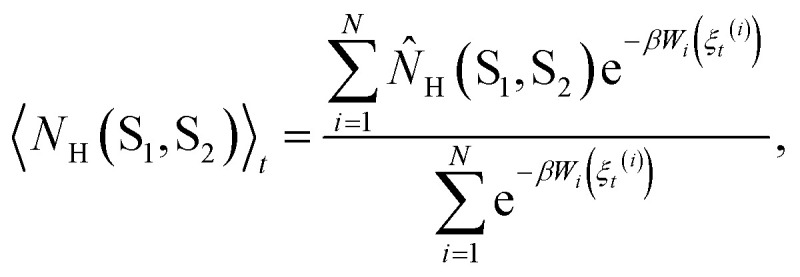
where the hydrogen bonds between the groups at each point along the trajectory are2b
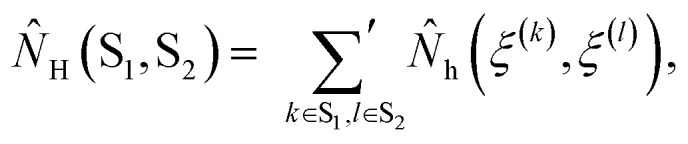
where *N̂*_H_(*ξ*^(*k*)^, *ξ*^(l)^) is 1 if *ξ*^(*k*)^ and *ξ*^(*l*)^ are hydrogen bonded positions and 0 otherwise. The prime in the sum excludes the case that *k* = *l*. Let S_P_ and S_W_ denote the sets of oxygen atom positions in the peptide and water solvent, respectively. The average number of intrapeptide hydrogen bonds is determined by 〈*N*_H_(S_P_, S_P_)〉_*t*_, and the average number of hydrogen bonds between peptide and solvent is determined by 〈*N*_H_(S_P_, S_W_)〉_*t*_. Averages for interaction energies are computed analogously to eqn (2) with *N̂*_H_(S_1_, S_2_) replaced by the corresponding sum of interaction energies between the residues in S_1_ and S_2_.

### D. Simulation details

All molecular dynamics simulations of the peptides discussed in Section IVB were performed using NAMD^37^ with the CHARMM27 force field.^38–40^ Several point mutations of the WT trpzip1 were performed. After each mutation, the peptides were equilibrated in vacuum for one nanosecond. Each peptide was solvated using *N*_w_ ≈ 5700 TIP3P water molecules in a square cuboid solvent box with two equal sides of length *L*_*xy*_ ≈ 47 Å, and a longer side along the *z*-axis of length *L*_*z*_ ≈ 82 Å. The systems were then ionized for neutrality using Na^+^ or Cl^−^ ions as implemented in the NAMD protocol for ionization. In E5L, three Cl ions are added, in K8L there is one Cl ion, and in the Trp to Ser mutants there are 2 Cl ions.

After ensuring neutrality, the systems are equilibrated for 1 nanosecond in explicit solvent using NVT conditions. In each equilibration procedure, the C_α_ of the first and twelfth residues are held fixed on the *z*-axis. After equilibration, the peptides were analyzed using the NAMD plug-in Timeline for secondary structure stability and calculation of the root mean squared deviations. Secondary structure analysis of the solvated structure was carried out to ensure the stability of the β-sheet motif after equilibration.

During the ASMD simulations, the peptides are stretched along the *z*-axis. The reaction coordinate is defined as the distance between the C_α_ of the first and twelfth residues. The C_α_ of the first residue is held fixed, while the harmonic steering potential is applied to the twelfth residue. At the start of the first stage of each simulation, the distance between the stationary and pulled atom is constrained to 4 Å. This constraint ensures that the peptide does reach a minimum at approximately 4.7 Å during the pulling simulation. All equilibrations and simulations are carried out at a temperature of 300 K.

For each peptide, the PMF is obtained using a pulling velocity of 1 Å ns^−1^ and a sampling size of 100 trajectories per stage, over the entire ASMD simulation of 20 evenly partitioned stages. The peptides are stretched for 40 Å for a total reaction coordinate, *r*_ee_, of 4 to 44 Å.

In all equilibrations and simulations, the Langevin thermostat is used to maintain the temperature of the “bath”, the van der Waals interaction cutoff distance is 12 Å, the smooth-switching function begins at 8 Å, and electrostatic forces are computed using the Particle-Mesh Ewald method with a grid size of <1 Å. Equilibration begins by first undergoing energy minimization to remove unfavorable contacts. This is accomplished by minimizing for 100 000 steps using the conjugate gradient method. A damping coefficient of 5 ps^−1^ with a decay period of 100 fs and a damping time constant of 50 fs was used. The NVT Ensemble is used to equilibrate the system for 1 ns and for all subsequent simulations.

### E. Root mean square deviations

In Section IVB, the Root Mean Square Deviations (RMSDs) are compared for each mutation at four different values of *r*_ee_, 12, 16, 24, and 28 Å. These distances were selected based on the PMF curves obtained from simulation in this work, and to be presented among the results of Section IVB. Snapshots of the structures of each mutant at those distances were obtained from an average structure from the individual trajectories. Next, the structures were aligned using the RMSD Calculator Plugin available in the VMD package.^20^ The structures were aligned using only the backbone of all of the residues (*i.e.* 1 through 12). The RMSDs were calculated one at a time using the WT structure and one mutant structure.

## III. Experimental methods

### A. Peptide synthesis

Peptides were synthesized using standard solid phase synthetic techniques by New England Peptide, Inc. Each peptide contained a standard NH_2_ N-terminal structure and an amidated C-terminal end. Molecular weight and purity were confirmed by mass spectroscopy and reversed phase HPLC. Typical peptide purity was >95%.

### B. Circular dichroism

Peptides were dissolved to a final concentration of 20 μM in 15 mM NaPO_4_ (pH 7.0) for far UV (190 to 250 nm) measurements. Spectra were obtained as a function of temperature on an Applied Photophysics Chirascan spectrophotometer utilizing a 1 mm pathlength quartz cuvette. Melting curves were obtained in both UV regions at 1 °C intervals after a 5 minute incubation at the new temperature with an averaging time of 5 seconds. Thermal denaturation was fully reversible as evidenced by recovering approximately 98% of the CD signal upon cooling and by the observation that reverse and forward melting curves were superimposable. Raw CD data was converted to mean molar ellipticity according to:3
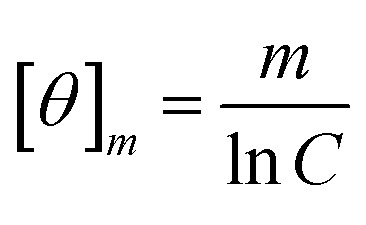
where *m* is the raw CD reading in millidegrees, *l* is the cell path length in millimeters, *n* is the number of amino acids in the peptide, and *C* is the micromolar concentration.

CD signal at 228 nm (far UV) was used to visualize the loss of CD signal as a function of temperature and to qualitatively compare thermal stabilities of all the peptides while not considering the mechanism of thermal denaturation. Raw CD signal in the region of 5 to 8 °C was used to calculate an average CD value that was arbitrarily set to 100% CD signal. Percent remaining CD signal was then calculated and plotted *versus* temperature. Finally, the data presented represent the average of three independent experiments, all of which were superimposable to within 0.25 °C of *T*_m_. Only the loss of CD signal are reported for the peptides because the exact experimental unfolding pathway of the WT peptide is unknown and a simple inspection of the CD signal *versus* temperature curves indicates that the effects of individual mutations on the unfolding pathway are pleiotrophic.

## IV. Results

### A. Experimental determination of peptide stability

**Fig. 2:**
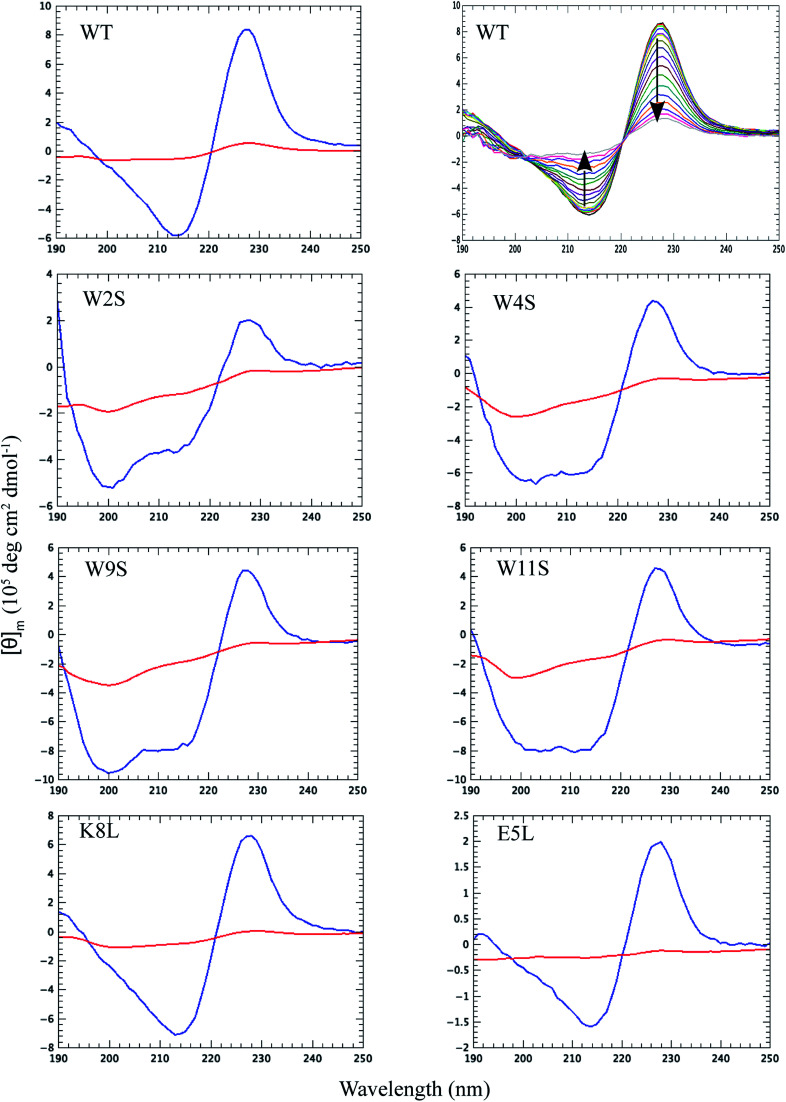
Far UV CD spectra for the peptides at 5 °C (blue lines) and at 90 °C (red lines) as noted in the upper left corner of each panel. The second wild type panel illustrates the full far UV spectrum of the WT peptide as a function of temperature between 5 and 90 °C plotted every 5 °C. The temperature increase is marked by a loss of ellipticity at 228 nm and 212 nm, the direction of which is shown by the arrows in the second panel. The global analysis to determine the peptide multi-wavelength melting temperature was performed between the 200 nm inflection point and 250 nm.

Thermal CD is a powerful technique for monitoring the change in secondary structure as a function of temperature.^41–43^ The far UV CD spectra of the peptides are shown in Fig. 2. The spectrum is characterized by a positive CD peak at approximately 228 nm that decreases in magnitude as a function of increasing temperature. Simultaneously, there is an increase in the magnitude of the negative CD signal at 213 nm. The thermal folding–unfolding reaction is reversible (refer to Fig. S8 in ESI†) and cooperative, the spectrum is characterized by a high signal to noise ratio, and the thermally induced transition can be effectively monitored exclusively at 228 nm exciton-coupled band or by performing a global analysis using data between 200 nm and 250 nm.

**Fig. 3:**
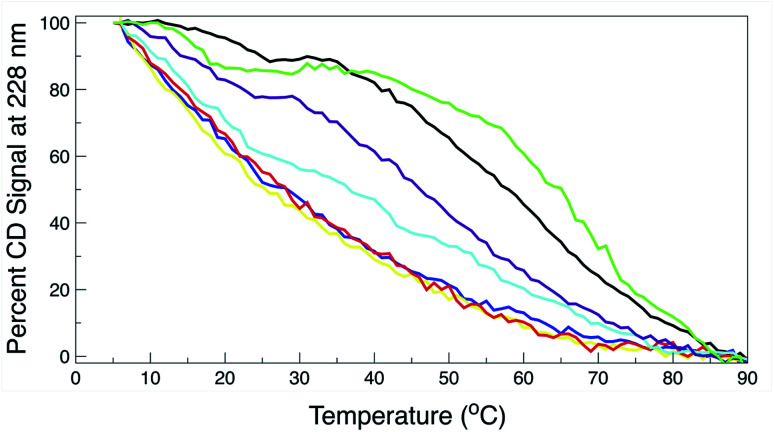
Percent remaining CD signal at 228 nm as a function of temperature for the trpzip WT and mutant peptides. Line colors for all graphs: wild type, black; W2S, cyan; W4S, red; W9S, yellow; W11S, blue; K8L, purple; E5L, green. The curves represent the average of three independent determinations. The standard deviation for the percent of remaining CD signal between the three readings at any temperature was less than or equal to 2%.

The thermal behavior and far UV CD profile of the peptides is similar to data presented on other tryptophan zipper peptides by Cochran *et al.*,^2^ and on other trpzip mutants.^44^ Peptide unfolding transitions are independent of peptide concentration and the forward and backward unfolding curves are superimposable. Simple visual inspection of the thermal unfolding curves in Fig. 3 reveals generally broad thermally induced transitions, with some curves perhaps exhibiting two state behavior (*e.g.*, WT and K8L) while other peptides are either more complex (*e.g.*, E5L and W2S) or are characterized by a nearly continuous thermal transition (*e.g.*, W4S, W9S and W11S). Because the unfolding curves are apparently more complex than a simple two state model (N ↔ U), no attempt has been made to deconvolute the curves for this work. Instead, it is possible to simply rank order the relative stabilities by inspecting the overall curves and/or calculating the temperature at which 50% of the overall CD signal at 228 nm is lost. Qualitatively all the curves can be compared to the WT curve. WT peptide denaturation exhibits an initial region of CD signal stability between 5 and 20 °C, followed by a transition period CD signal loss to 27 °C, a plateau region to 35 °C, a monotonically decreasing region to 85 °C, and a final plateau to 90 °C. Peptide K8L most resembles the WT curve only with the transition temperatures generally occurring several degrees before the WT peptide transitions. Peptide E5L is characterized by a significant thermal denaturation plateau between 20 and 37 °C. The peptide is less stable than WT at temperatures below 40 °C, but is more stable beyond that temperature. WS2 denatures without the initial plateau, but is characterized by two distinct slopes; a more rapid denaturation curve to 25 °C and a more gradual transition to 80 °C. The thermal denaturation curves for W4S, W9S, and W11S are superimposable. So in general, the effects of the single tryptophan substitutions are more destabilizing than are the single leucine replacements.

The absolute magnitude of the molar ellipticity at the 212 nm CD minimum and the 228 nm exciton varies between WT trpzip and its mutants, as reported in Fig. 3. The reason for this is unknown but may be the result of mutation induced changes to interactions in the peptide core or the degree to which tertiary structure may be altered. Nevertheless, the stability of a given structure can be discerned from the percentage loss of signal. By visual inspection of the denaturation curves of the percent remaining CD signal (Fig. 3), the relative stability of the trpzip peptides at 50% loss of signal (at 228 nm) in this work is: (WT ∼ E5L) > K8L > W2S > (W4S ∼ W9S ∼ W11S). Looking at the temperature at which 50% of the CD signal is gone, is much like how an inhibitory concentration IC_50_ is calculated. From the data shown in Table 1 (also independent of any assumed unfolding pathway) a relative stability order is revealed: E5L > WT > K8L > W2S > (W4S ∼ W11S) > W9S.

**Table 1:** Temperature at which 50% of the 228 nm CD signal is lost during the thermal denaturation of WT and mutant trpzip peptides. Numbers in parentheses represent the standard deviation of three experiments

Peptide	Temperature (°C)
WT	58.1 (0.3)
W2S	36.5 (0.3)
W4S	28.1 (0.2)
W9S	25.5 (0.4)
W11S	28.1 (0.3)
K8L	46.1 (0.2)
E5L	64.9 (0.1)

### B. Computational energetics of the chosen peptides

**Fig. 4:**
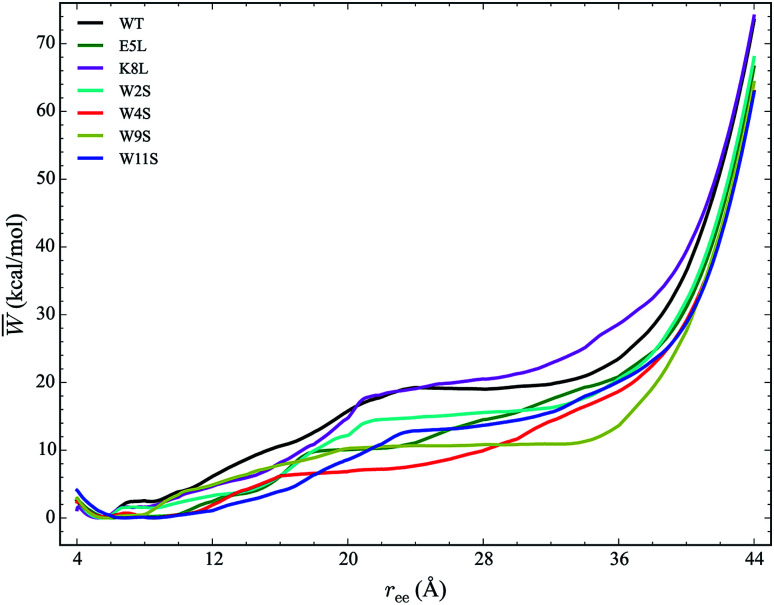
PMFs for each peptide in explicit water solvent with free energies reported relative to the minimum for each curve. The WT PMF is shown in black. The mutant PMFs are shown for comparison. Each PMF is obtained at a velocity of 1 Å ns^−1^ with a sampling size of 100 trajectories per stage.

**Fig. 5:**
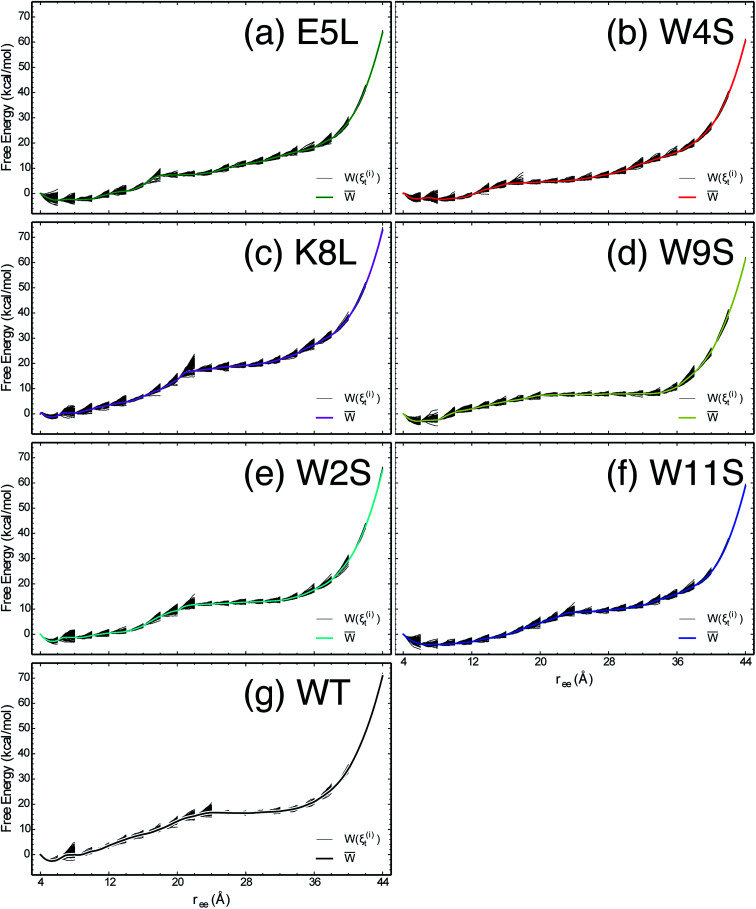
The PMFs shown in Fig. 4 are reproduced here for the seven peptides as indicated in each panel, overlaid by the work along each of the nonequilibrium trajectories which expand within each stage and contract at the beginning of the subsequent stage.

The work required to unfold the peptide varies along the unfolding pathway. This variation is expressed as the PMF for a specified reaction coordinate, namely the end-to-end distance *r*_ee_. The energetics of the mechanical unfolding of the WT trpzip1 and six mutants in explicit solvent are shown in Fig. 4. The PMFs were obtained using the ASMD method with 100 trajectories per stage, at a pull velocity of 1 Å ns^−1^. We have shown previously,^19^ that a pulling velocity of 1 Å ns^−1^ is sufficient for convergent and reliable determination of the overall trend in energetics between peptides. The accuracy of the method was verified by ensuring that the distribution of work values across all trajectories within a given stage was limited to less than 5 kcal mol^−1^ as illustrated in Fig. 5. This is sufficient as long as there is a single dominant trajectory about which the nonequilibrium trajectories distribute uniformly. When this assumption fails, the MB-ASMD method^45^ can be used to improve the sampling of the ensemble. Meanwhile, the error accumulates from stage to stage as the projection at the end of each naive ASMD adds an error bar on the order of the standard deviation of the final energies. We used only the naive ASMD method in this work because it is computationally simpler (and cheaper), and provides enough accuracy to obtain qualitative comparison particularly within the first 10 Å when the accumulated error from only a few stages is still small.

In Fig. 4, the PMF of the WT peptide is shown in black. The mutations substantially affect the magnitude and overall curvature of the PPMFs, shown in Fig. 4, indicating a difference in stability due to mutation. The mutations exhibit both stabilizing and destabilizing trends in the energetics. In particular, the K8L mutant (magenta curve), is the only one which results in a PMF that closely resembles the PMF of the WT peptide. For the first 20 Å of the pull, the WT peptide is the most stable until K8L surpasses the WT to become the most stable. This indicates that K8L maintains stability similar to that of the WT or even exceeding that of the WT. The E5L mutation is substantially destabilizing and differs from the observed behavior for K8L. This is perhaps surprising because both mutations directly effect the salt bridge formation between residues Glu5 and Lys8.

Additional general observations about the energetics of the mutations can be made from Fig. 4. All peptides, except W11S (blue), have a similar minimum near 5 Å. The W11S mutant has a very broad, not well-defined minimum. In the WT peptide, near 24 Å, the PMF peaks and begins to plateau. Interestingly, in every mutant, except for W11S, this “peak” is shifted to the left. Also, for the first half of the pull, the W11S mutant maintains the lowest PMF. Two of the mutants, E5L and W2S, show signs of a second “minimum” near 14 Å. Overall, it is clear that the mutations induced different energetic behaviors of the peptides during mechanical stretching simulations.

One useful metric for analyzing the trends in the peptide stability is the particular values of the free energies Δ*A*(*N*, *V*, *T*) equal to the PMF at given extensions of *r*_ee_ relative to the minimum value of the PMF—*cf.*Fig. 4. The experimental stability ordering generally follows the simulation rank order. As listed in Table 2, at *r*_ee_ = 4 Å, the order of Δ*A* values is W11S > W9S > W2S > E5L > WT > W4S > K8L. This stability order indicates that in the first stages of the ASMD pull, the Trp to Ser mutants are all stabilizing as measured by the Δ*A* of the PMF. At *r*_ee_ = 12 Å, the ordering in the trend of the Δ*A* values inverts and the Trp to Ser mutants are generally less stable than the other peptides (with the exception of W9S): WT > W9S > K8L > W2S > E5L > W4S > W11S. It should also be noted that the WT peptide becomes the most stable peptide. At *r*_ee_ = 20 Å, the WT remains the most stabilized peptide with K8L as a close second: WT > K8L > W2S > W9S > E5L > W11S > W4S. At 28 Å, there is a turnover between WT and K8L with K8L becoming the most stabilized peptide, which is consistent for the remainder of the pull: K8L > WT > W2S > E5L > W11S > W9S > W4S. Overall, the last three sets of trends remain very similar. The only differences between the trends at 28 Å and 36 Å are the relative orderings of E5L and W2S, and W4S and W9S: K8L > WT > E5L > W2S > W11S > W4S > W9S. At the end of the reaction coordinate, at 44 Å, the order of the peptides is: K8L > WT > W2S > E5L > W9S > W4S > W11S. Again, K8L and WT remain the most stable peptides while W9S, W4S and W11S are the least stable. The order of the stability of the first four peptides is the same as the order at 28 Å. The only difference between the two trends, at 28 Å and at 44 Å, is that the W9S and W11S are reversed.

**Table 2:** The Δ*A* (in units of kcal mol^−1^) values at structurally significant distances of *r*_ee_ (denoted across the top) for each peptide (on left side) based on the PMF curves from Fig. 4

*r*_ee_ (Å)	4	12	20	28	36	44
WT	2.55	6.12	15.73	19.02	23.50	73.43
W2S	2.81	3.29	12.17	15.57	20.54	67.94
W4S	2.37	1.93	6.84	9.93	18.70	62.94
W9S	2.91	4.86	10.28	10.85	13.60	64.27
W11S	4.09	1.09	8.60	13.68	20.13	62.84
E5L	2.71	2.44	10.07	14.52	20.84	66.56
K8L	1.23	4.65	14.71	20.52	28.61	74.06

**Fig. 6:**
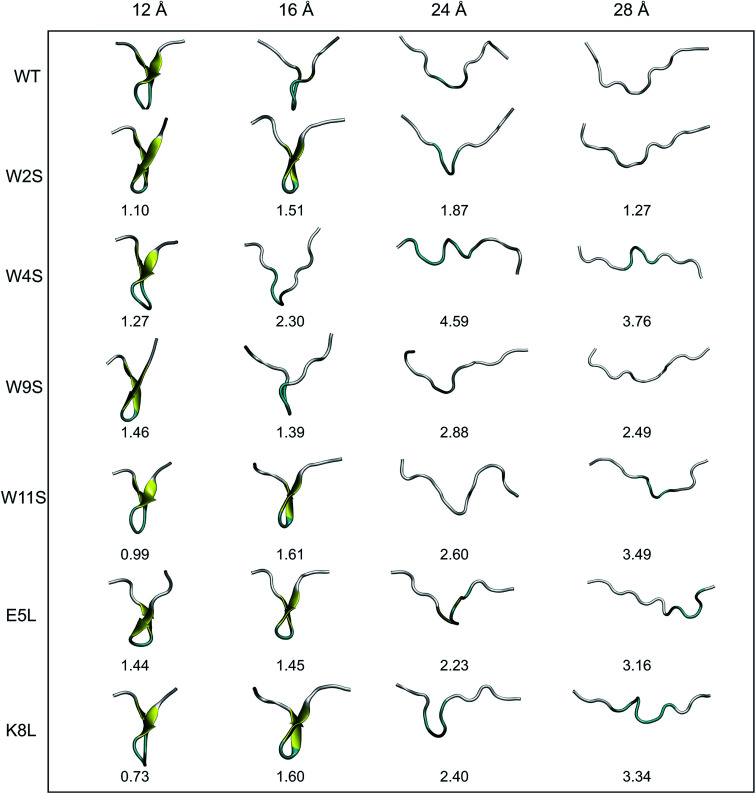
Illustrations of the unfolding pathways of each peptide. Each structure, taken from the end of a given ASMD stage, comes from the trajectory whose work best matched the Jarzynski average. The peptides are colored by their representative secondary structures motifs. The coloring scheme is the following: yellow represents β-hairpin structure, cyan corresponds to turn structure, and white is random coil structure. The RMSD values obtained, in Å, are listed below each structure. The RMSDs are calculated between the WT structure and a mutant structure at the corresponding stretching distance (top).

Another useful metric for discussing the effect of the mutations on the overall structural changes and pathways the peptides undergo during forced unfolding, is the calculation of Root Mean Square Deviations (RMSDs). The RMSDs provide a comparative tool to determine how different the secondary structure is between the WT and the mutant peptide. Snapshots of each peptide were taken along the trajectory at specific values of *r*_ee_ (*i.e.* 12, 16, 24, 28 Å). These values of *r*_ee_ correspond to features observed in the PMFs in Fig. 4. The snapshots of the structures and the corresponding RMSD obtained for that structure *versus* the WT are shown in Fig. 6. Overall, all of the peptides maintain the β-hairpin structure for the first 8 Å of the pull. The *r*_ee_ with the narrowest distribution of RMSD values sampled occurs at 12 Å. At that *r*_ee_, K8L has the smallest RMSD value, 0.73 Å, which indicates that the mutant structure is most similar to the WT structure. This reinforces findings that the K8L mutant is most similar in stability to the WT peptide. At 12 Å, the mutants with the largest deviations (*i.e.*, those least similar to the WT) are W9S and E5L. It can also be observed that the WT, W4S and W9S mutants begin to lose their β-hairpin structure at 16 Å. The broadest range of RMSD values sampled occurs at the next *r*_ee_ of 24 Å. Notably, the largest deviation from the WT structure at 24 Å occurs with the W4S mutant, which has an RMSD value of 4.59 Å. It should also be noted that at 24 Å, no peptide exhibits any residual β-hairpin structure. At a *r*_ee_ of 28 Å the RMSDs also cover broad range from 1.27 (W2S) to 3.76 Å (W4S). At this distance, which is more than halfway through the reaction coordinate, several of the mutations retain structure characteristic of the turn. Specifically, the W4S, W11S, E5L and K8L structures are still being stabilized by a few remaining pair interactions between residues that had stabilized the β-hairpin.

### C. Comparison of hydrogen bonding profiles

**Fig. 7:**
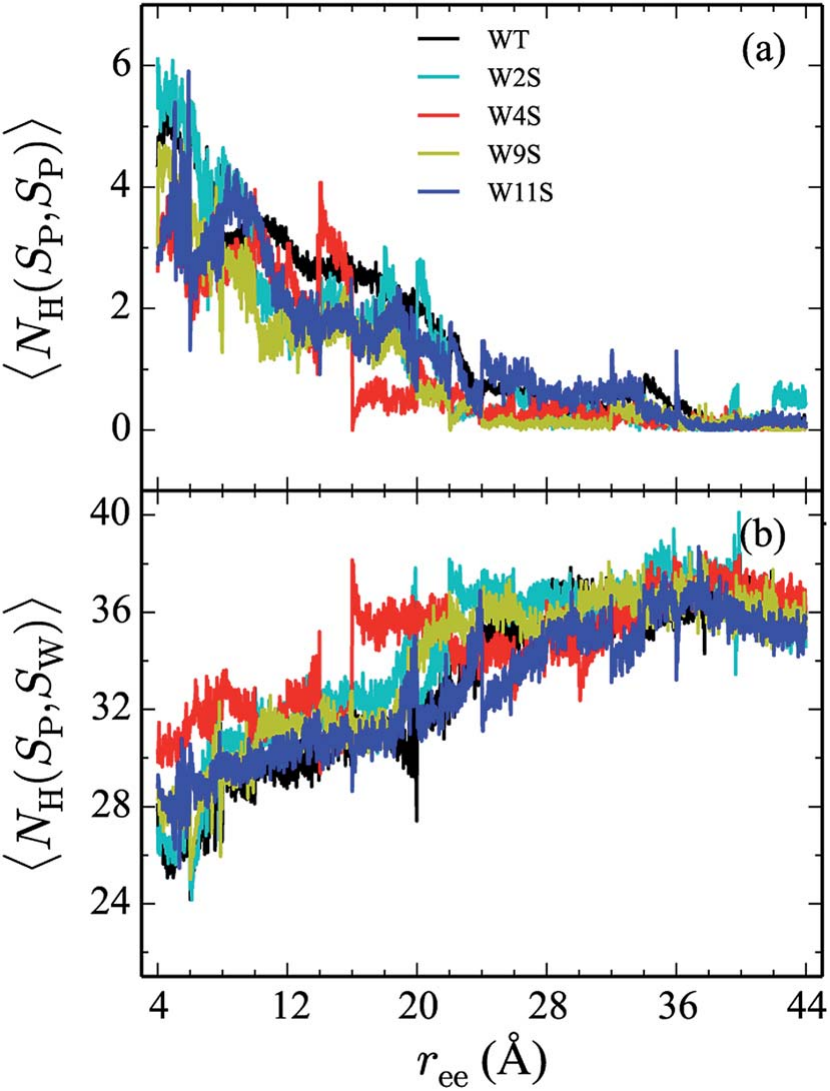
The weighted average number (*cf.*eqn (2)) of hydrogen bonds for trpzip1 WT (black) and the Trp to Ser mutants (at positions 2, 4, 9 and 11 noted in the legend) in explicit solvent are shown. The weighted average number of hydrogen bonds formed within the peptide are shown in panel (a) and the bonds between the peptides and the explicit water solvent is shown in panel (b). For each panel, the average is of 100 trajectories per stage at pulling velocity of 1 Å ns^−1^.

**Fig. 8:**
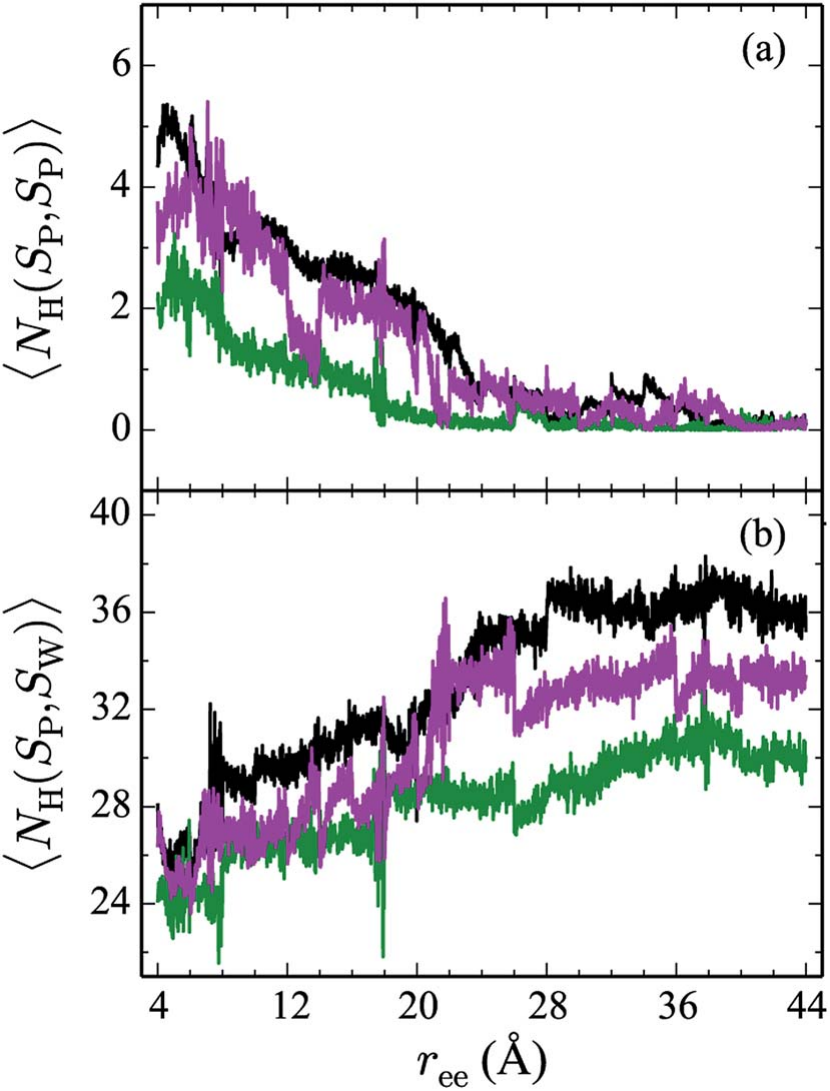
The weighted average number (*cf.*eqn (2)) of hydrogen bonds for trpzip1 WT (black) and the salt bridge mutants, E5L (green) and K8L (magenta), in explicit solvent are shown. The weighted average number of hydrogen bonds formed within the peptide are shown in panel (a) and the bonds between peptides and the explicit water solvent is shown in panel (b). For each panel, the average is of 100 trajectories per stage at pulling velocity of 1 Å ns^−1^.

Hydrogen bonds have been shown to play a significant role in the determination of pathway and structure of peptides.^9^ As shown in Fig. 7, the mutation of the tryptophans to serine does not substantially effect the hydrogen bonding patterns in explicit solvent. However, the salt bridge mutations, E5L and K8L, affect both the hydrogen bonding patterns within the peptide and between the peptide and explicit water solvent as shown in Fig. 8. In panel (a), the initial mutant structures have fewer bonds than the WT peptide. The WT begins with 5 intrapeptide hydrogen bonds, K8L begins with 3, and E5L begins with 2. At a *r*_ee_ of 28 Å, the mutants and the WT intrapeptide bonds taper off to 0 bonds. The largest difference in the patterns is shown in panel (b). Though the peptides begin with about 24 bonds to solvent, by the end of the unfolding pathway the E5L mutant (green) forms 6 fewer bonds to solvent than the WT peptide (black). That is, 29 total H-bonds for the WT peptide (5 intermolecular, 24 to solvent), 27 for K8L (3 intermolecular, 24 to solvent), and 26 for E5L (2 intermolecular, 24 to solvent). This trend is also evident in the fully unfolded state where the WT peptide has 36 H-bonds with the solvent compared to 32 for K8L and 30 for E5L. Thus there are more unsatisfied H-bonds for E5L in the unfolded state (6 compared to WT) than there are in the folded state (3 compared to WT). So although H-bonding is important to overall stability (as measured by Δ*A*), the entropic contributions are clearly predominant during unfolding such that the overall energetics favor WT over E5L. As K8L is closer to the WT peptide in H-bonding numbers, the pattern of enthalpy–entropy compensation must be similar to WT than it is (or the WT peptide is) to E5L. These results highlight the interplay between all of the intermolecular forces when determining how peptides are stabilized.

The Trp-to-Ser mutants all begin with circa 26 to 31 hydrogen bonds to solvent and, end with nearly 36 contacts to water as shown in Fig. 7b. This range is, on average, more than the WT and salt bridge mutants. At *r*_ee_ = 20 Å, the K8L and E5L contacts to solvent begin to differentiate. By the end of the pull, the WT peptide has 36 bonds to solvent, K8L has 33, and E5L has 30. Overall, the hydrogen bond patterns are not as sensitive to the serine mutants, but they are sensitive to the mutations of the salt bridging residues.

### D. Comparison of residue pair interaction energies

**Fig. 9:**
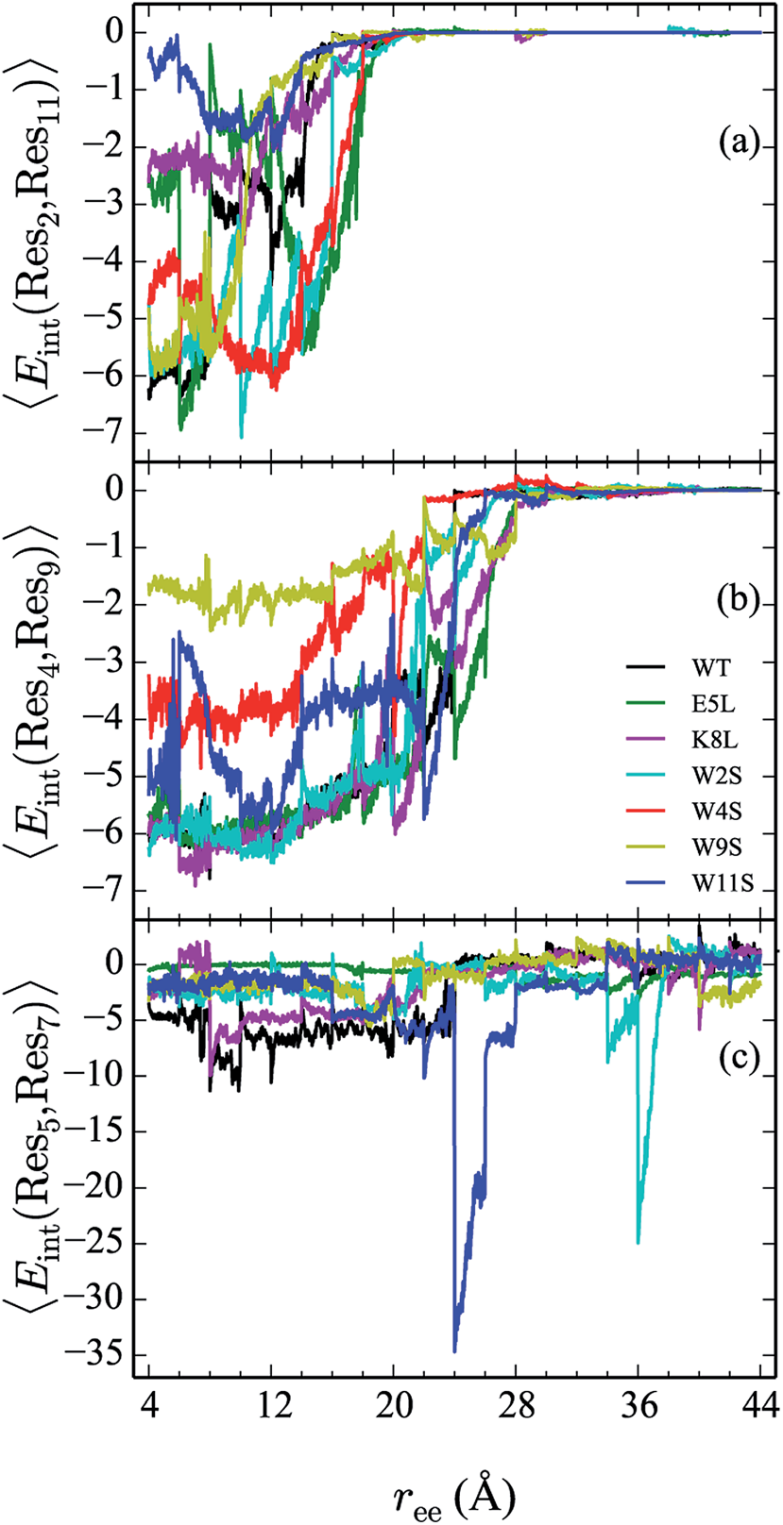
Comparison of the 2–11 (a), 4–9 (b), and 5–7 (c) interactions across all peptides. The energies (in kcal mol^−1^) for each curve are obtained using a weighted average of 100 trajectories per stage at a velocity of 1 Å ns^−1^.

**Fig. 10:**
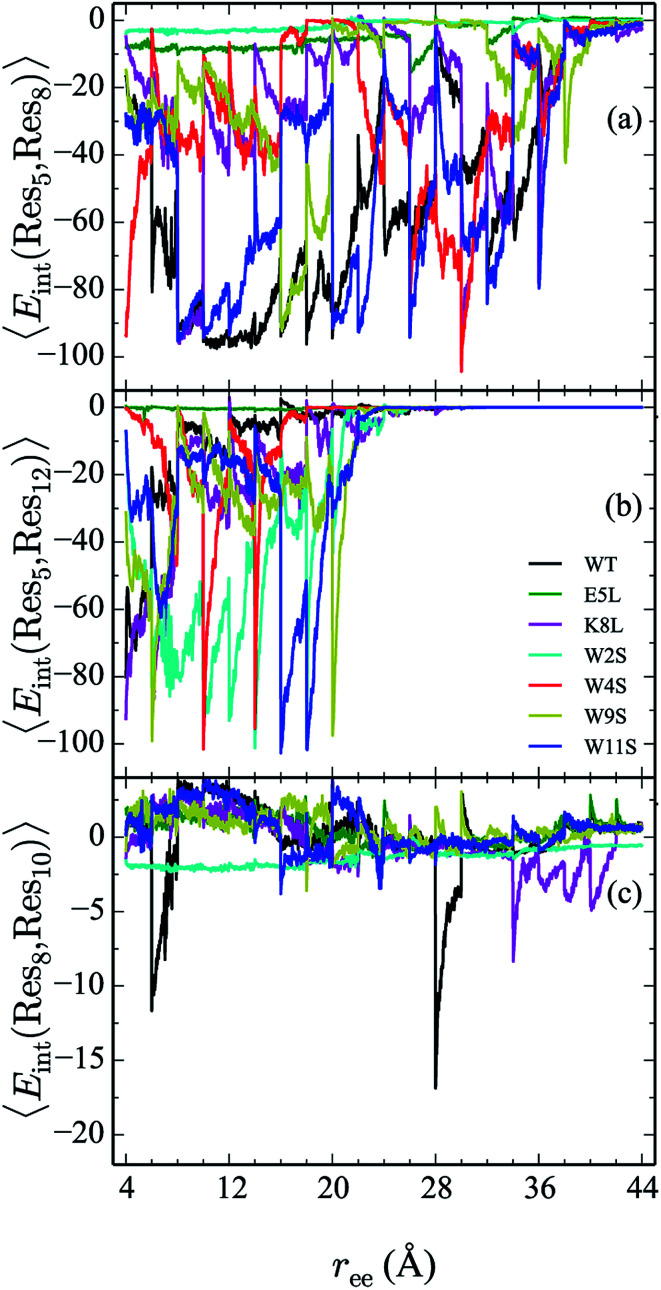
Comparison the 5–8 (a), 5–12 (b), and 8–10 (c) interactions across all peptides. The energies (in kcal mol^−1^) for each curve are obtained using a weighted average of 100 trajectories per stage at a velocity of 1 Å ns^−1^.

**Fig. 11:**
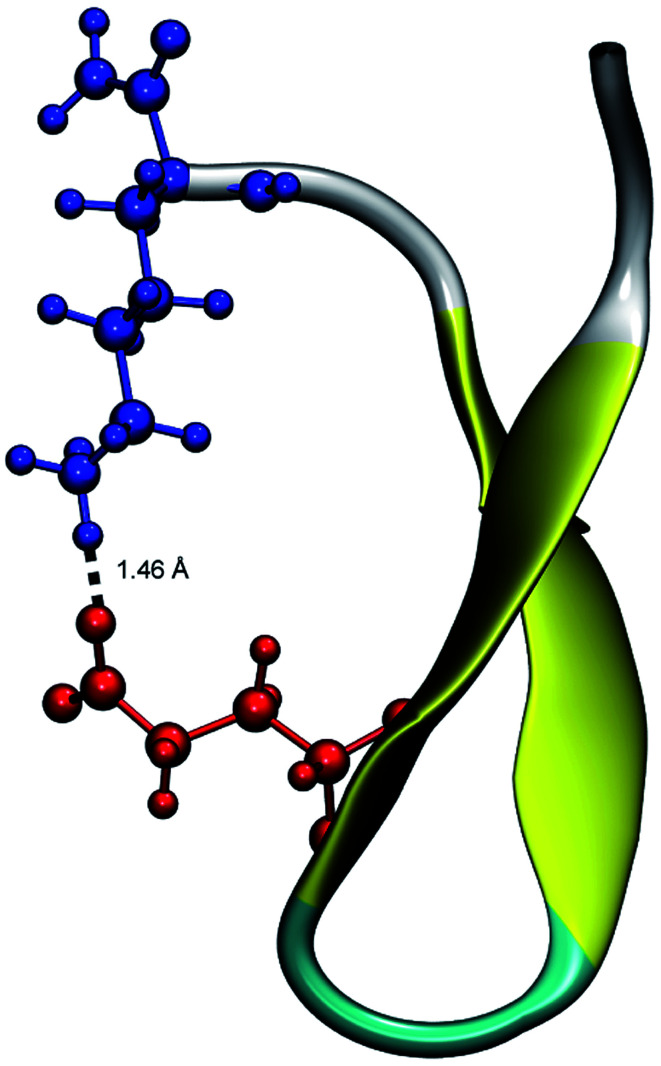
Unweighted average structure of K8L depicting the stabilizing interaction between Glu5 (red) and Lys12 (blue) seen in Fig. 10. The distance between the residues is shown as a dashed black line and was measured to be 1.46 Å. The overall *r*_ee_ of this selected structure is approximately 8.6 Å which is just at the distance where the 5–12 interaction is breaking and just before the overall K8L PMF begins to rise more steeply.

From the calculation of the persistence of specific residue pairs within each peptide, we can determine an approximate ordering for when the contacts become non-interacting. The WT interaction energy curves, shown in black in Fig. 9 and 10, have been reproduced from our previous work.^19^ As noted earlier, the profiles exhibit discontinuities at and sometimes in-between the transitions between stages resulting from the projection of the structures at the end of each naive ASMD stage. As such, the scatter in the data is significant as is provides an indication of the range of interaction energies across the pulling coordinate. As observed in that prior work, the most stabilizing interaction for the WT peptide in Fig. 10a is the Glu5–Lys8 pair interaction. However, according to the residue pair interaction energies obtained for the salt bridge mutants, E5L and K8L, there is a competing salt bridge within K8L. As shown in Fig. 10b (magenta curve), the most stabilizing interaction within the mutant peptide K8L is Glu5–Lys12. The 5–12 interaction breaks at an overall end-to-end distance *r*_ee_ near 8.6 Å. A representative structure of the simulated ensemble a this *r*_ee_ position and with the 5–12 distance at only 1.46 Å apart is shown in Fig. 11. The Glu5–Lys12 interaction is stronger within K8L because the Glu5–Lys8 interaction is no longer available as a result of the mutation of Lys8 to Leu8. In this mutant, the terminal lysine residue can interact with the glutamic acid, helping to stabilize the peptide during the stretch. This interaction is not possible in the E5L mutant because the negatively charged Glu has been mutated to a neutral Leu. In the WT peptide, the interaction is present but is not dominant because Lys8 is positioned closer to Glu5.

Due to the stabilizing nature of the competing salt bridge within the K8L mutant, its unfolding pathway mimics that of the WT peptide. The Glu5–Lys12 is stabilized at the beginning of the pull at approximately −80 kcal mol^−1^ and tapers off to 0 kcal mol^−1^ at a *r*_ee_ of 24 Å. This coincides with the implications of the experimental observed WT and K8L CD curves in Fig. 3. Their qualitative structure is nearly identical, with similar plateaus (see temperatures 25–35 °C) and downward slopes (see temperatures 10–20 °C and 30–60 °C), except the K8L loss of CD signal is displaced downwards (meaning that at any given temperature, K8L experiences more lost CD signal than does the WT peptide). This reflects in the overall lower K8L *T*_m_ (temperature at 50% loss of CD signal).

The Glu5–Lys12 interaction is also an example of a nonnative contact formed spontaneously during the course of unfolding. Its subsequent rupture adds to the work required to stretch the peptide. The position of the charged residues within the peptide is known^22,23^ to control the stable structure adopted by the peptide; they can be favorable (resulting in a salt bridge) or detrimental (diminishing hairpin formation). On the other hand, cross-strand stabilization by sidechain–sidechain interactions in a similar secondary structure, protein GB1, has been seen^46^ to not be the most dominant factor contributing to folded structures.

The interaction between residues at the 2 and 11 positions is shown in Fig. 9a. In the WT, the dominant interaction arises from one of the two Trp–Trp interactions. Though the peptides range in stability from −1 to −6 kcal mol^−1^—values that generally indicate slight stabilization,—it is apparent that the mutations lead to discernible trends in the stabilization. For example, the W11S mutant (blue curve) shows the most dramatic change in energy arising from the 2–11 interaction. This is expected because the mutation of the tryptophan to serine at the 11 position directly effects that pair. For each peptide, the interactions taper off near 20 Å.

The interaction energies between residues at the 4 and 9 positions, shown in Fig. 9b, were seen to obey a similar pattern. In the WT, this interaction is also one of the two Trp–Trp interactions. The W4S (red curve) and W9S (yellow curve) mutants exhibited more diminished interaction energies for the 4–9 interactions. This finding is consistent with what was expected since those mutants were mutated to directly effect that residue pair interaction. All of the other peptides exhibit stabilization at −6 kcal mol^−1^. As expected the interactions are maintained for a longer period of time than the Trp2–Trp11 interactions and taper off near 28 Å. The interactions between residues at the 8 and 10 positions shown in Fig. 10c, do not significantly contribute to the stability of the peptide. This is to be expected because in the WT, the interaction between Lys8 and Thr10 is unfavorable. However, the WT peptide does show slight stability with the Lys8–Thr10 pair at *r*_ee_ between 6–10 Å and 28–32 Å. Interestingly, the K8L mutant also shows slight stabilization near the end of the pull between the Leu8–Thr10 residue pair at *r*_ee_ between 34–44 Å. Profiles for the other mutant peptides do not show any stabilization. Similarly, the interaction between residues 5 and 7, as shown in Fig. 9c, only slightly contributes to stability of the peptide for all mutants. There is a slight spike in the stability of −35 kcal mol^−1^ for the Glu5–Asn7 in the W11S mutant at *r*_ee_ between 24–28 Å.

## V. Discussion

The interplay of intermolecular forces and the determination of a predominating stabilization force has been studied in many systems. The balance between backbone hydrogen bonds, side chain interactions, and stability of the turn region of the 16-residue hairpin fragment of protein G assessed from molecular dynamics trajectories led to the conclusion that the side chain interactions are the most sensitive to perturbations in the system, while the hydrophobic core was very stable, and the hydrogen bonds were least stable.^47^ In addition to affecting the stability and monomeric folding–unfolding transition of a peptide, mutations have been shown to influence the aggregation behavior of model systems.^48^ This includes not only the overall formation of aggregates, but the kinetics of aggregate formation. These effects have been shown to result from a combination of changes to hydrophobicity, charge, and secondary structure propensity. Dodson and coworkers^48^ observed that mutations can either increase or decrease aggregation rates. Unlike previous arguments, they suggested that the difference in aggregation rates does not result from specific side chain interactions, but because the mutations effect the overall “physiochemical properties of the system”.

We found no experimental evidence in this work that the mutations alter the tertiary of the trpzip peptides. Consequently, the mutations simply alter the folding–unfolding transition through the ability of an unfolding peptide (either thermally or by mechanical pull) to compensate for this process. These compensatory interactions arise from the formation of salt bridges or H-bonding interactions (either intrapeptide or with water). Thorpe and coworkers^49^ used a constraint-based model to investigate the formation of nonnative contacts within the mechanical unfolding of several mutated proteins of varying sizes and secondary structures. However, they were unable to discern whether mutations had a substantial effect on their observables.

The central result of this work is the resolution of the residue effects on the structure and stability of WT trpzip1 due to the mutations illustrated in Fig. 1 using experimental and computational approaches. The ASMD simulations provide near peptide-scale information about the structure and energetics for the steered unfolding. The spectroscopic information obtained from experiments as a function of temperature provides a trove of structural macroscopic data—*e.g.*, the loss of secondary structure or tertiary contacts. Comparison of between the computational and experimental quantities is necessarily indirect of the differences in the scales accessible to each. An additional caveat to the comparison between the experiments and simulations, lies in the fact that the computational results obtained here are approximate because of the use of naive ASMD to obtain the profiles in energy, hydrogen bonding and other observables along the pulling coordinate. The collapse of the nonequilibrium ensemble onto a single configuration at the beginning of each new stage leads to quantitative errors. Namely, configurations that are energetically comparable to that of the chosen configuration, but that are far away structurally would not be sampled. Their exclusion thus leads to an overestimate of the free energy, but these are comparable for the family of mutants under consideration here. As such, the differences in their energies which are the basis of the discussion of relative trends holds. Hence it is not surprising that the absolute rank order of stability is not identical between CD experiments and the ASMD simulations. The relative stabilities correlate better as the pull progresses, a phenomenon that is not completely understood, but is a function of the complex structure in the ASMD PMF curves.

Observables, such as hydrogen-bonding, are in principle more sensitive to the errors of naive ASMD because they depend on the structure of the particular structures sampled in the ensemble. The fact that hydrogen-bonding converged more readily in the smaller peptides used to benchmark our earlier work^17,24,25^ is indicative that for such peptides, that the PMF is determined by a single dominant pathway in the nonequilibrium ensemble. In the present set of peptides, the hydrogen bond profiles exhibit discontinuities at junctures between stages. This is a cause for alarm because it indicates that the most favored configuration—in the sense that the work required to pull it out matched the average work—is not necessarily characteristics of the structure of all of the likely configurations in the ensemble. In the present case, the subsequent pulling of this configuration brings back some—if not all—of the alternative configurations as seen in the relaxation of the hydrogen-bond profiles with a stage and the fact that subsequent projections can fall on configurations with associated with a different dominant trajectory—as exhibited by a different value in the hydrogen-bonding. While these particular peptides appear to be described by branched nonequilibrium trajectories, they are only mildly branched in the sense that the naive ASMD trajectory samples them within each stage. The peripatetic curves seen for the hydrogen-bonding profiles thus provide a sense of the error at small differences in *r*_ee_ while the overall shape of the profile is a good approximation of the hydrogen-bond changes at large differences in *r*_ee_.

The behavior of the trpzip mutants is complex from either an experimental or from a simulation point of view, let alone from a combined perspective. Far UV CD is a powerful technique for measuring the energetics of peptide/protein unfolding. In this instance however, a full thermodynamic analysis of the data is frustrated by the complex (and non-uniform) thermal unfolding curves. None of the mutants exhibit ideal two state (N ⇌ D) behavior and in fact the actual unfolding pathway may differ between the mutants with different or multiple intermediate states (N ⇌ I ⇌ D). However it is still possible to rank order the stabilities of the peptides in a manner completely analogous to how the ASMD experiments are analyzed. In future work on this system that determines the actual unfolding pathway and the accompanying energetics, it will be possible to directly compare free energies that are determined either experimentally or through simulation. It was surprising that single mutants in the trpzip system had such wide-ranging effects. Some of this complexity is seen in the simulations and some is not. Thus we have taken steps towards providing a framework that relates experimentally determined unfolding and the simulation of mechanical unfolding in a model system. One goal that remains is to better account for the complexity that is seen in the macroscopic behavior of the system under thermal transition and the atomistic detail that is uncovered in the simulations.

A second central result of this work is the confirmation that the ASMD method can be used to obtain trends in structure and stability across a family of related peptides. ASMD measures the mechanical force required to unfold a peptide. In that sense it is analogous to atomic force microscopy measurements of protein unfolding (for a review see ref. 50) albeit in a different solvent environment. In both techniques, it may be difficult to fully compare the free energy of unfolding along the forced reaction coordinate (and tethered ends) with the values obtained experimentally in free solution. Few studies have directly compared AFM force–extension curve results (or SMD/ASMD simulations) with thermal or chemical denaturation in solution. In some systems, not only are atomic force microscopy unfolding landscapes rate and force dependent, they differ from the thermal unfolding landscape.^51^ In other systems, there is much better quantitative agreement between the techniques.^52,53^ A direct and meaningful comparison between free energy landscapes is further complicated in that the magnitude of the free energy differences is reduced with slower pulling rates.^54^ We too have seen this effect in previous ASMD studies.^19^ While there is no *a priori* reason why mechanical stability should be correlated to thermodynamic stability, ASMD (and earlier SMD) numerical experiments have been seen to be predictive of the qualitative equilibrium behavior. In general, differences in the free energy landscapes are more pronounced in larger protein systems than they are for smaller peptides (with simple secondary structure motifs) or smaller proteins with simpler tertiary structure arrangements. That is why in this report we do not make quantitative comparisons between the two techniques. Instead, we focused on a simple single secondary structure motif containing peptide. A goal of this work is to evolve ASMD into a technique that can be fully correlated to the thermodynamic stability of any protein system. The use of the more expensive (and less approximate) full relaxation ASMD, where all the structures at the end of a given stage are fully re-equilibrated before the next stage, may lessen the magnitude of the free energy differences.

Both experiment and simulation qualitatively identify the same top 3 and the bottom 3 stable mutants. In the simulations, these are identified according to the energetic ordering of the peptides at 4 Å of pull where they are slightly compressed from the initial structures. It is interesting to note that the most stable peptides (wild type and K8L) begin the ASMD simulation as the least stable in this sense. This result perhaps indicates that the less stable peptides have an increased structural plasticity to accommodate the initial end-to-end compression. The absolute rank order (compared to the far UV CD stability order) improves as the pull progresses. The reason for this is not clear, but it may indicate that the ASMD simulations allow for a sampling of structural states that affect the stability of a mutant at a given *r*_ee_ (Å), but that are spectroscopically silent. In future work, we will include simultaneous comparison to biophysical techniques other than CD (or in addition to CD) in order to better understand what each experimental technique is capable of seeing along the ASMD reaction coordinate. For example, hydrogen–deuterium exchange may provide a direct correlation to H-bonding patterns measured in the ASMD simulations. Alternatively, one could use enhanced sampling techniques^55^ to characterize the free energy of alternate order parameters characteristic of the unconstrained unfolding that to have a more direct analog of the process taking place in the CD experiments.

E5L is an intriguing mutant and appears to have a heterogeneous thermal unfolding path. Experimentally it behaves completely differently than the other peptides. At temperatures between 5 and 20 °C, the fraction of unfolded proteins increases faster than the WT and in way that is comparable with the other mutations. After the intermediate transition (at 20 to 40 °C), the fraction of unfolded proteins increases slower than all of the other proteins, suggesting the presence of some stabilizing interaction. This is qualitatively seen in the structure of the E5L PMF. Although it is not immediately apparent, the differences in H-bonding patterns along the reaction coordinate appear to correlate with the trends in relative stability in the structure of the intermediate seen during UV CD thermal denaturation.

## VI. Conclusion

In this work, we have investigated six mutants of trpzip1 using experimental and computational methods to determine the stability of each peptide. We compare the computationally determined mechanical unfolding pathways of each mutant with the WT peptide. We have also calculated the side chain–side chain and hydrogen bonding effects within each mutant. From this analysis, we have found that the stability of the competing salt bridge between Glu5–Lys8 and Glu5–Lys12 within K8L is substantial and directly effects the energetics and unfolding pathway of the peptide.

The mutations E5L and K8L affect the degree of hydrogen bonding between intrapeptide and peptide–solvent bonds. We found evidence in the computed unfolding pathways to suggest that there is an interplay between hydrophobic collapse and side chain–side chain interactions. This is also confirmed from the experimental CD spectra and thermal unfolding. Like the WT peptide, K8L is stabilized by a salt bridge. However, this contact is between the Glu5 and Lys12 residues, which is a nonnative contact in the WT peptide. In the case of E5L, such a contact is not possible because the Glu at the fifth position has been mutated to a leucine residue. Thus the K8L mutant is the most stable because structurally and energetically it resembles the WT peptide.

In our previous article,^19^ we explored the energetics of unfolding two small β-hairpins, trpzip1 and chignolin, employing ASMD sampling the interactions of a protein specified by the CHARM27 force field to obtain energetics and pathways. In the present work, we now see that this computational approach can also be used to discern between mutants in a systematic study of trpzip1. The energetics of the mechanical unfolding of a set of trpzip1 mutants allow for the identification of the most critical contacts stabilizing not only trpzip1 but the entire unfolding pathway. The trends are benchmarked by the accompanying experimental results, while detailed information in the computations allow for a better understanding of their residue-specific origin. Thus this work serves both to provide a deeper understanding of trpzip stability and a proof-of-concept for the use of ASMD in mutation assays.

## Conflicts of interest

There are no conflicts to declare.

## Supplementary Material

RA-010-D0RA00920B-s001
