# Supplementary material for: The relative stability of trpzip1 and its mutants determined by computation and experiment
Source: RSC Adv. 2020 Feb 12;10(11):6520–35. doi: 10.1039/d0ra00920b (PMC9049704; doi:10.1039/d0ra00920b)
Supplement: RA-010-D0RA00920B-s001 [file RA-010-D0RA00920B-s001.pdf]

Electronic Supplementary Material (ESI) for RSC Advances.
This journal is © The Royal Society of Chemistry 2020

Supporting Information for "Relative stability between trpzip1 and its mutants determined by computation and experiment"

Hailey R. Bureau,[1] Stephen Quirk,[2] and Rigoberto Hernandez[1,*]

[1]*Department of Chemistry*
Johns Hopkins University
Baltimore, MD 21218
[2]*Kimberly-Clark Corporation, Atlanta, GA 30076-2199*

(Dated: January 19, 2020)

The Potentials of Mean Force (PMFs) obtained for the wild type, and shown in Fig. 5 of the main text, are reproduced here at a higher resolution so as to more clearly reveal the work trajectories at each stage. Specifically, Figs. S1- S7, illustrate the PMFs for wildtype (WT) trpzip1 and six mutants, E5L, K8L, W2S, W4S, W9S and W11S, respectively. Each figure shows the distribution of work trajectories calculated within stages of the Adaptive Steered Molecular Dynamics simulations. The distributions provide an estimate of the error within each stage.

Far UV CD spectra at 5°C at the beginning and end of a temperature cycle going to 90°C and back are shown in Fig. S8, illustrating reversibility. The low termperature result for all seven proteins is also included in the plots of Figure 3 in the main document.

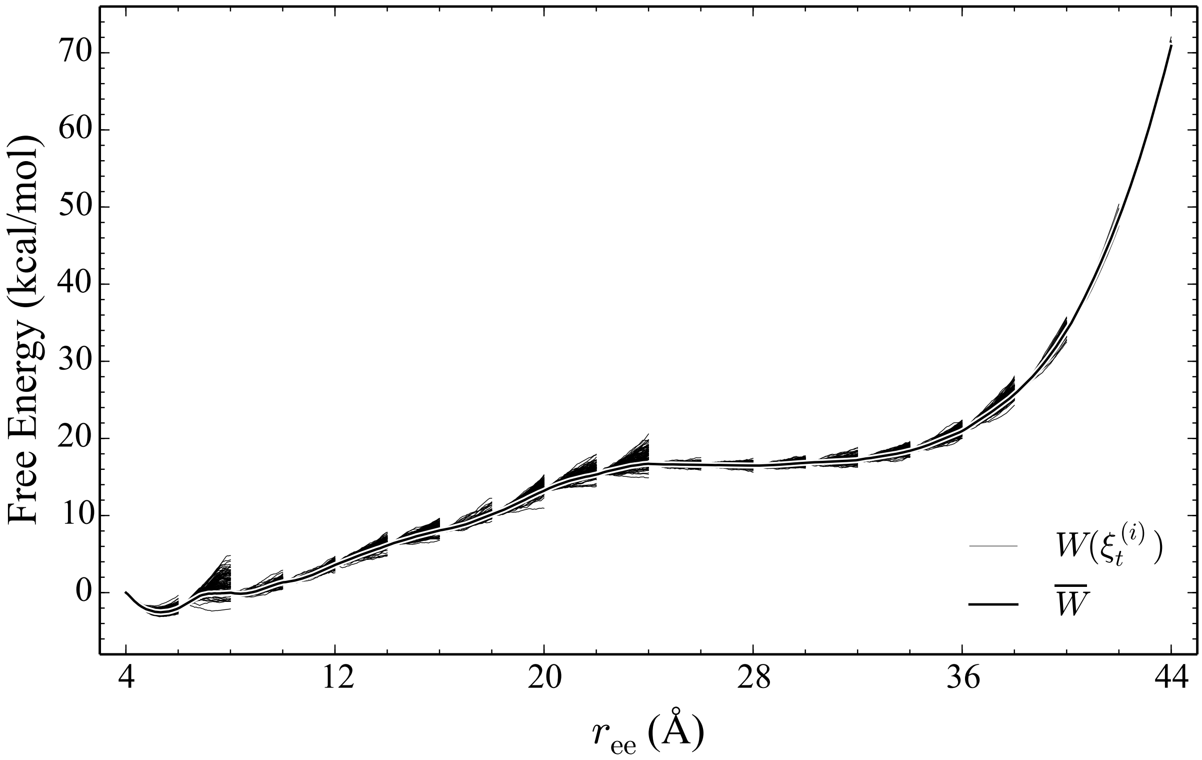

FIG. S1. PMF of WT calculated in explicit solvent using 100 tps at 1 Å/ns. The color scheme of the PMF corresponds to Fig. 5 of the submission. The black curves are the individual trajectories calculated per stage.

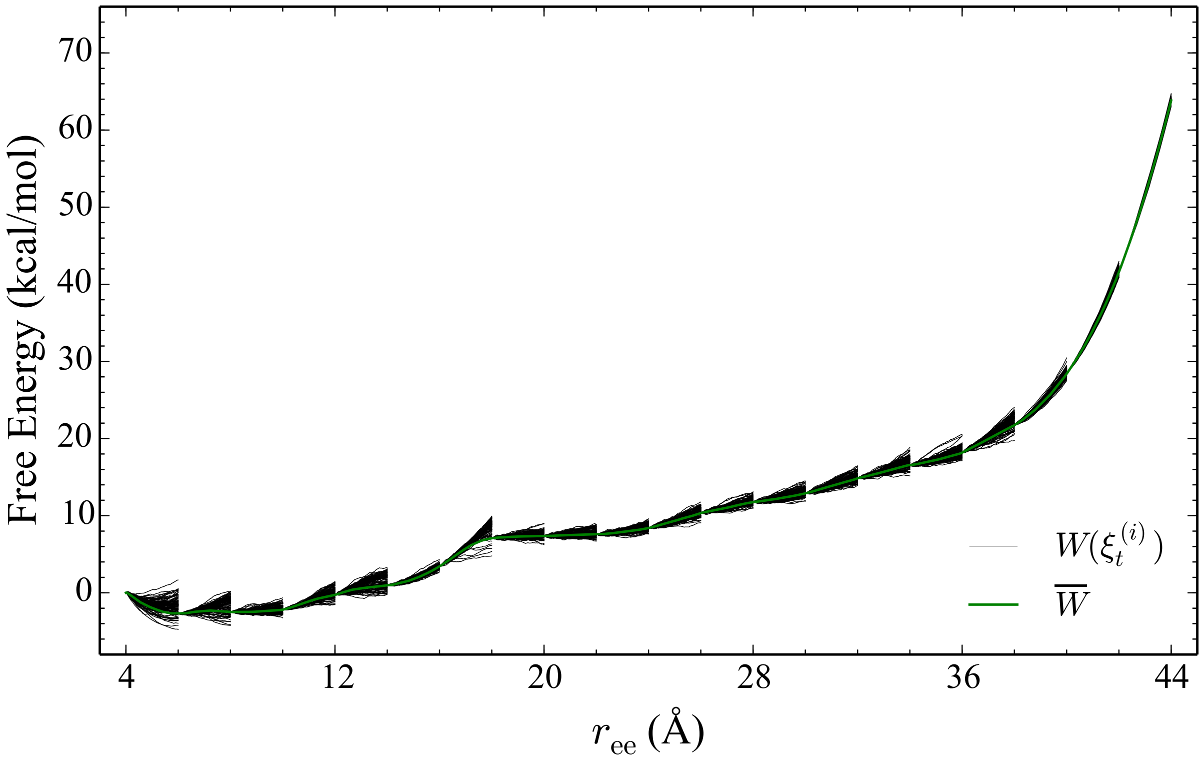

FIG. S2. PMF of E5L calculated in explicit solvent using 100 tps at 1 Å/ns. The color scheme of the PMF corresponds to Fig. 5 of the submission. The black curves are the individual trajectories calculated per stage.

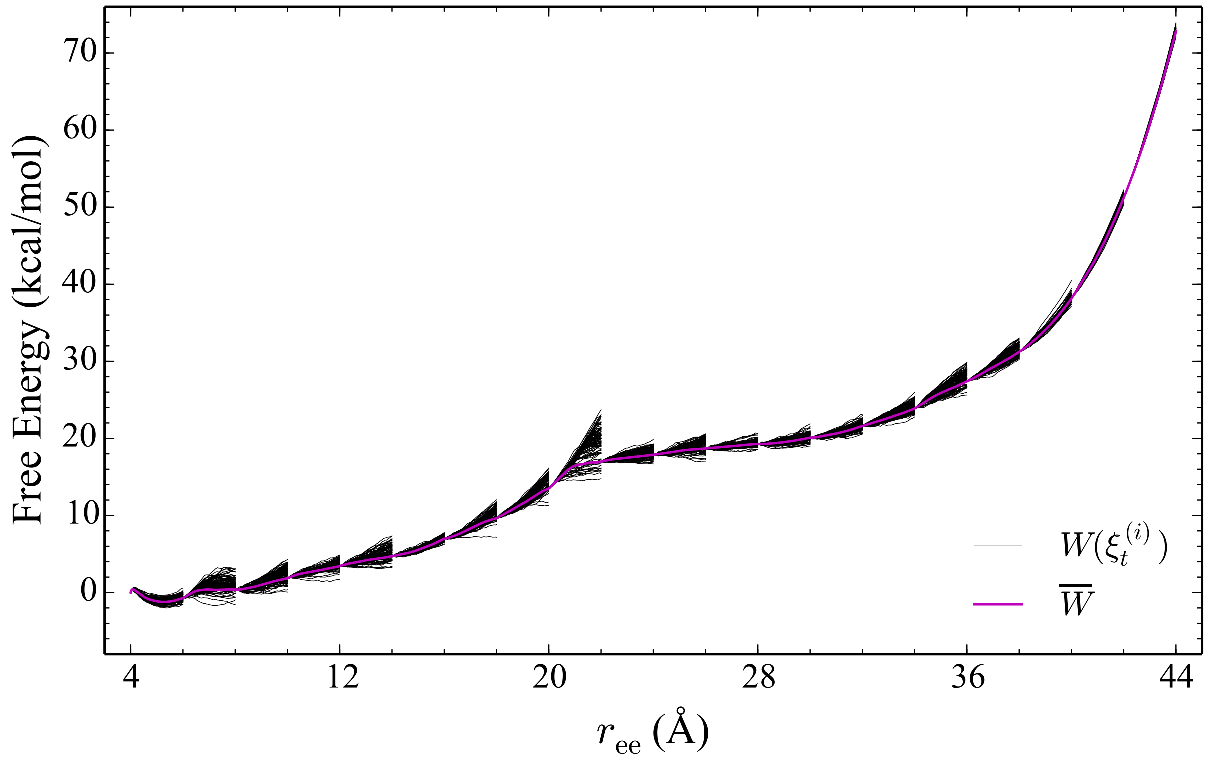

FIG. S3. PMF of K8L calculated in explicit solvent using 100 tps at 1 Å/ns. The color scheme of the PMF corresponds to Fig. 5 of the submission. The black curves are the individual trajectories calculated per stage.

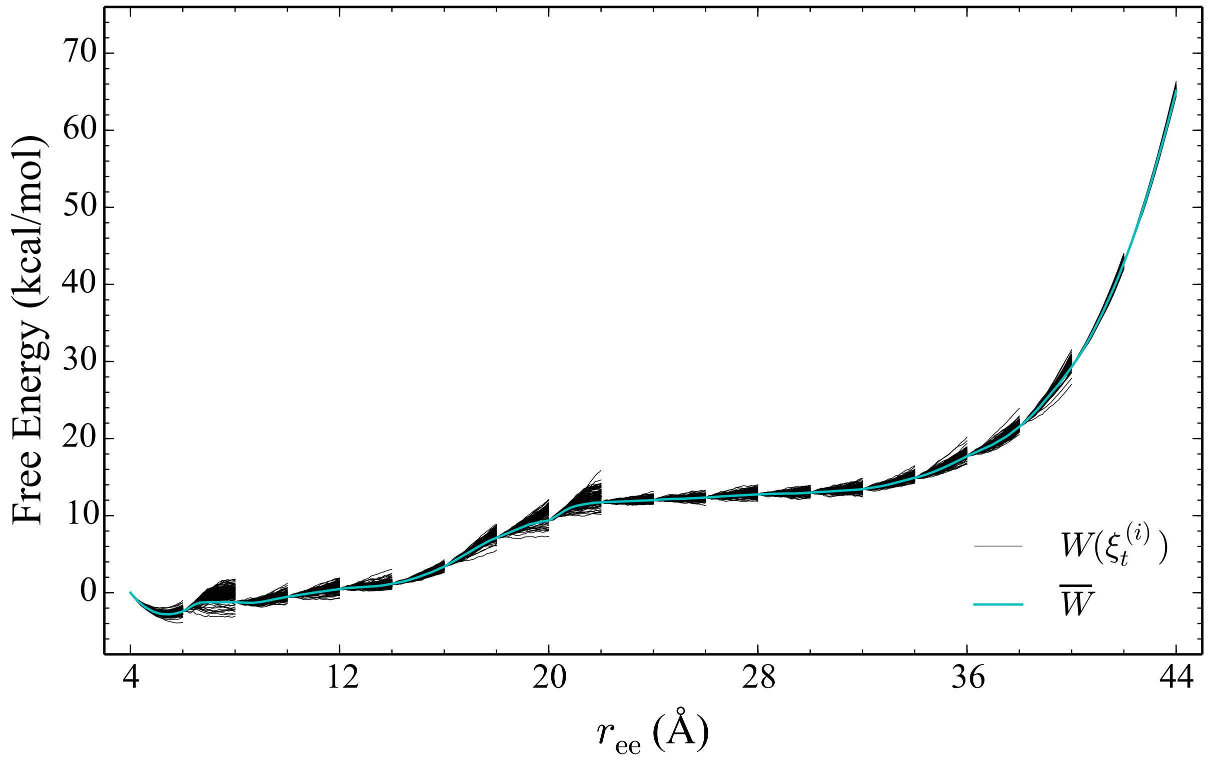

FIG. S4. PMF of W2S calculated in explicit solvent using 100 tps at 1 Å/ns. The color scheme of the PMF corresponds to Fig. 5 of the submission. The black curves are the individual trajectories calculated per stage.

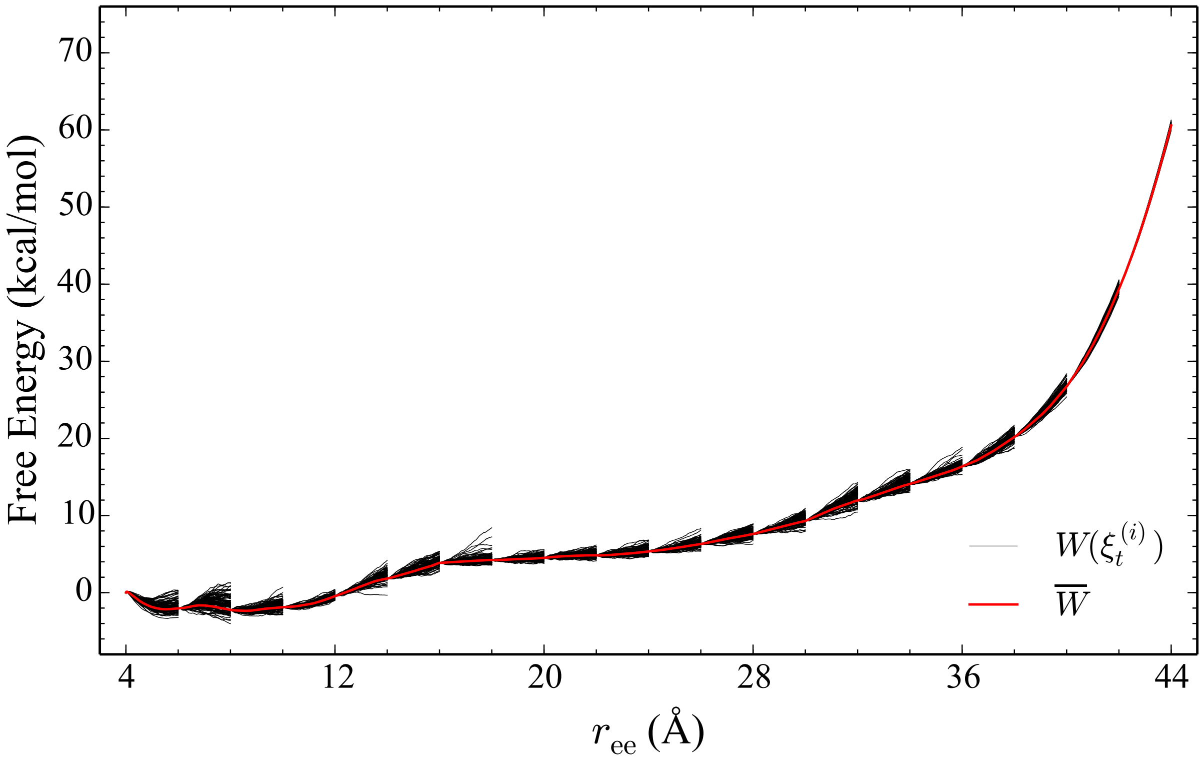

FIG. S5. PMF of W4S calculated in explicit solvent using 100 tps at 1 Å/ns. The color scheme of the PMF corresponds to Fig. 5 of the submission. The black curves are the individual trajectories calculated per stage.

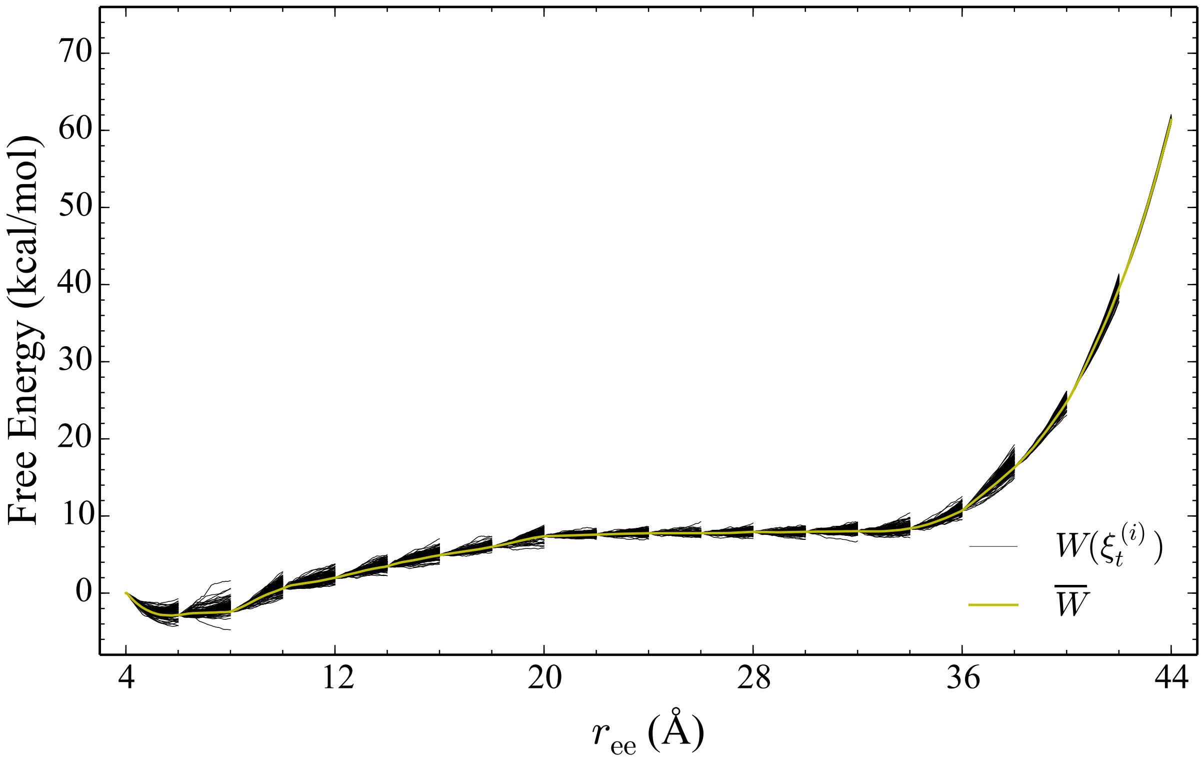

FIG. S6. PMF of W9S calculated in explicit solvent using 100 tps at 1 Å/ns. The color scheme of the PMF corresponds to Fig. 5 of the submission. The black curves are the individual trajectories calculated per stage.

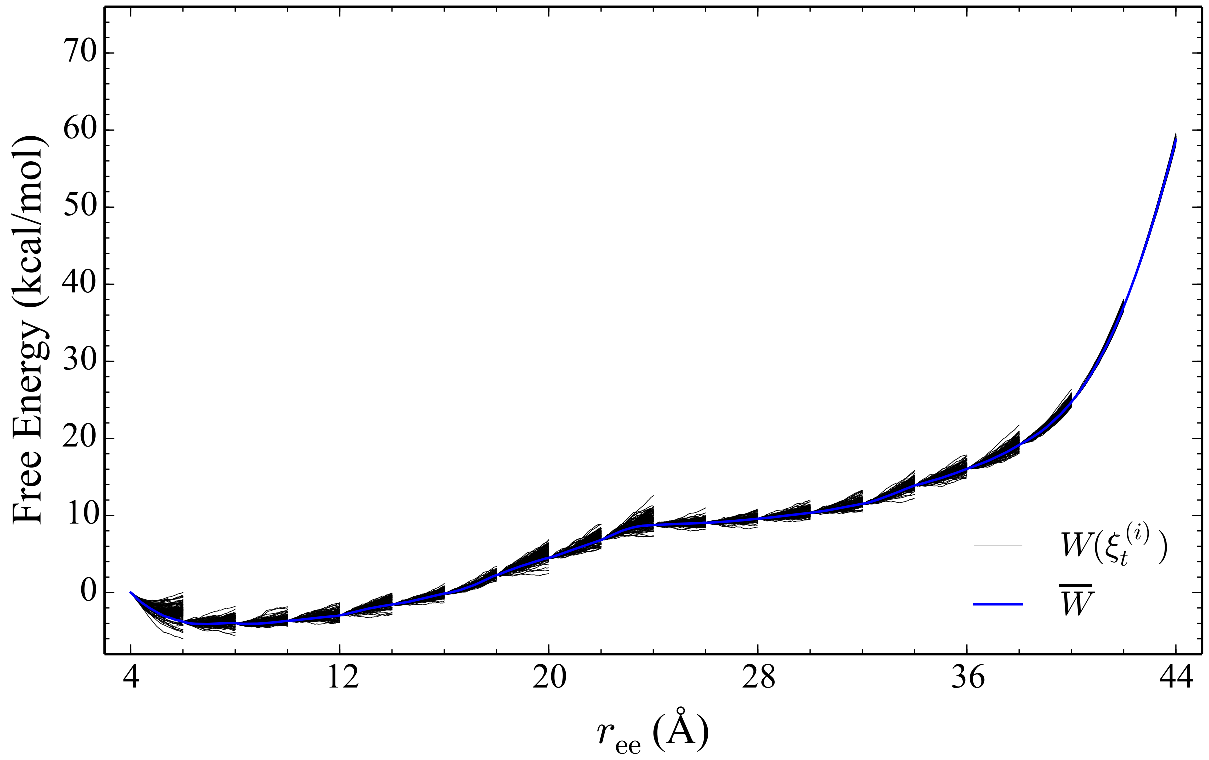

FIG. S7. PMF of W11S calculated in explicit solvent using 100 tps at 1 Å/ns. The color scheme of the PMF corresponds to Fig. 5 of the submission. The black curves are the individual trajectories calculated per stage.

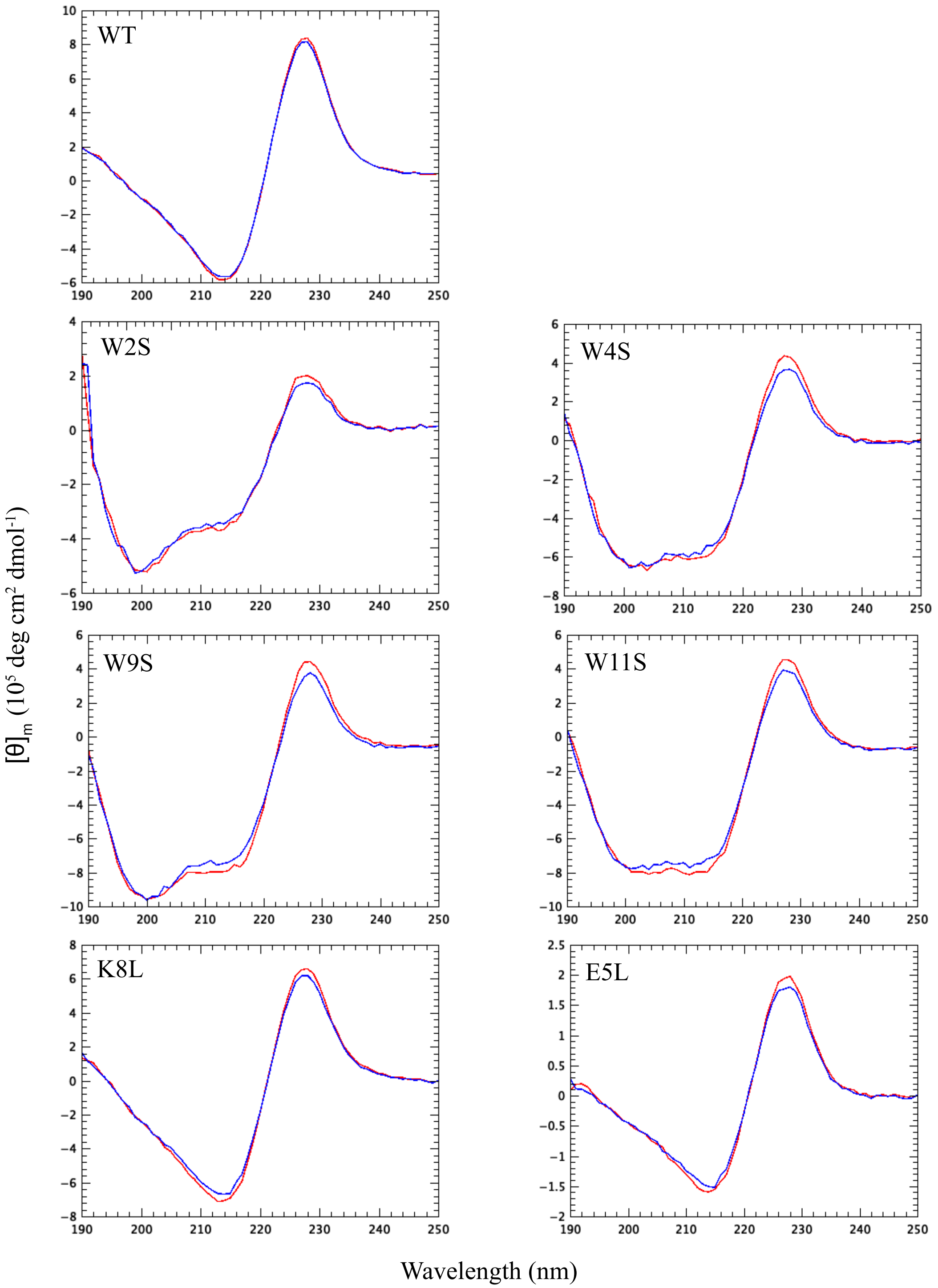

FIG. S8. Far UV CD spectra for wild type and mutant peptides at 5°C before the initial temperature ramp to 90°C (curves in blue) and after an identical ramp down (started immediately after the ramp up had finished equilibration at 90°C) from 90°C to 5°C (curves in red). All peptides exhibit reversible folding-unfolding reactions.
